# Retinal layers changes in patients with age-related macular degeneration treated with intravitreal anti-VEGF agents

**DOI:** 10.1186/s12886-023-03203-w

**Published:** 2023-11-13

**Authors:** Dan Zhou, Yan Hu, Zhongxi Qiu, Zirong Liu, Hongyang Jiang, Ryo Kawasaki, Jiang Liu

**Affiliations:** 1 School of Ophthalmology & Optometry, School of Biomedical Engineering, Wenzhou Medical University, Wenzhou, China; 2 Research Institute of Trustworthy Autonomous Systems and Department of Computer Science and Engineering, Southern University of Science and Technology, Shenzhen, 518055 China; 3 Department of Informatics, Osaka University Graduate School of Medicine, Suita, Osaka Japan

**Keywords:** AMD, PCV, Anti-VEGF, OCT, Retinal layers, Treatment response

## Abstract

**Background:**

The purpose of this study was to investigate retinal layers changes in patients with age-related macular degeneration (AMD) treated with anti-vascular endothelial growth factor (anti-VEGF) agents and to evaluate if these changes may affect treatment response.

**Methods:**

This study included 496 patients with AMD or PCV who were treated with anti-VEGF agents and followed up for at least 6 months. A comprehensive analysis of retinal layers affecting visual acuity was conducted. To eliminate the fact that the average thickness calculated may lead to differences tending to converge towards the mean, we proposed that the retinal layer was divided into different regions and the thickness of the retinal layer was analyzed at the same time. The labeled data will be publicly available for further research.

**Results:**

Compared to baseline, significant improvement in visual acuity was observed in patients at the 6-month follow-up. Statistically significant reduction in central retinal thickness and separate retinal layer thickness was also observed (*p* < 0.05). Among all retinal layers, the thickness of the external limiting membrane to retinal pigment epithelium/Bruch's membrane (ELM to RPE/BrM) showed the greatest reduction. Furthermore, the subregional assessment revealed that the ELM to RPE/BrM decreased greater than that of other layers in each region.

**Conclusion:**

Treatment with anti-VEGF agents effectively reduced retinal thickness in all separate retinal layers as well as the retina as a whole and anti-VEGF treatment may be more targeted at the edema site. These findings could have implications for the development of more precise and targeted therapies for AMD treatment.

**Supplementary Information:**

The online version contains supplementary material available at 10.1186/s12886-023-03203-w.

## Introduction

Vascular endothelial growth factor-A (VEGF-A) is known to play a crucial role in ocular angiogenesis, which is a predominant cause of blindness in various clinical conditions [1, 2]. The application of anti-VEGF therapy has revolutionized the treatment for many retinal diseases [3], including age-related macular degeneration (AMD), diabetic retinopathy (DR), retinal vein occlusion (RVO), retinopathy of prematurity (ROP) [4]. However, due to some adverse events and complications associated with anti-VEGF agents, a portion of patients fail to respond optimally to the therapy. [5–7]. Consequently, early identification and characterization of specific patterns are vital to predict treatment response and consider alternative treatment options [8].

AMD is currently the third leading cause of blindness worldwide, neovascular AMD (nAMD) is a subtype that causes progressive loss of central vision and severe visual impairment. Due to the choroidal neovascularization (CNV) of nAMD, anti-VEGF therapy has become its standard treatment. [9, 10]. Polypoid choroidal angiopathy (PCV), a choroidal vascular disease characterized by type 1 neovascularization associated with abnormal vascular networks and polypoidal lesions [11], is regarded as a subtype of nAMD [12, 13]. Optical coherence tomography (OCT) is widely utilized in the diagnosis of AMD and PCV because it helps clinicians visually see the structure of the retina and choroid [14]. Therefore, the changes in OCT images could be used to evaluate the effect of anti-VEGF therapy [15, 16].

Neovascularization occurring in nAMD most commonly originates from the choriocapillaris, manifesting as various degrees of exudation in retinal pigment epithelium (RPE) and intraretinal fluid on OCT [17]. Although AMD is generally considered a disease of the outer retina, much research has shown that both inner and outer retinal layers change during disease progression [18–21]. However, retinal alterations after intravitreal anti-VEGF therapy for AMD are typically assessed in terms of visual acuity (VA) or central subretinal thickness (CST) in previous studies [12, 22], with little emphasis on changes in individual retinal layers or only a single layer such as retinal nerve fiber layer (NFL) or ganglion cell layer (GCL) [23, 24]. Besides, some studies have found that features such as retinal thickness, retinal vascular density, and RPE cell shape show different manifestations between the fovea and other regions in patients with AMD, and may occur in different ways as the disease progresses, affecting visual function [25–27]. However, there is no publicly available dataset with a large amount of labeled data for analysis. Accordingly, this study aims to segment retinal layers in OCT images of patients with AMD or PCV who underwent anti-VEGF therapy to explore potential improvements in individual retinal layers and conduct a subregional assessment of the retina to study the treatment response of different regions against VEGF agents. We will make our labeled data publicly available.

## Methods

The present study utilized an open dataset provided by the Asia Pacific Tele-Ophthalmology Society (APTOS),^1^ which was acquired from the Rajavithi Hospital of Thailand and the Aravind Eye Hospital of India. The dataset was labeled by the Zhongshan Ophthalmic Center of China, including anonymized patient information such as age, gender, eye side, and medical condition diagnosed by specialists. The dataset mainly consisted of five diseases, including nAMD, PCV, diabetic macular edema (DME), retinal vein occlusion (RVO), and cystoid macular edema (CME). The anti-VEGF agents patients accepted included bevacizumab, ranibizumab, aflibercept, conbercept, and VA measurements were converted to the logarithm of the minimum angle of resolution (logMAR) for statistical purposes. The data set provided OCT images with at least 6 directional scans before and after treatment for each patient. The CST was then marked and measured by doctors at Zhongshan Eye Hospital. Structural OCT was performed with the SPECTRALIS HRA + OCT imaging platform (Heidelberg Engineering, Heidelberg, Germany).

The inclusion criteria were as follows (1): aged greater or equal to 18 years; (2) diagnosed with nAMD or PCV; and (3) accepting consecutive monthly anti-VEGF therapy of aflibercept for 6 months. The exclusion criteria were as follows: (1) the presence of any other retinal diseases including diabetic macular edema (DME), retinal vein occlusion (RVO), and cystoid macular edema (CME) and so on; (2) low image quality caused by media opacities; or (3) an abnormal signal strength index of images. In this paper, the study focused on patients with nAMD and PCV who received bevacizumab considering the balance of data volume. Thus it led to the enrollment of 496 patients from 544 eyes, which comprised 324 eyes with AMD and 220 eyes with PCV, with a total of 8450 OCT images after the exclusion of patients with incomplete data and unclear or excessively noisy OCT images.

**Fig. 1:**
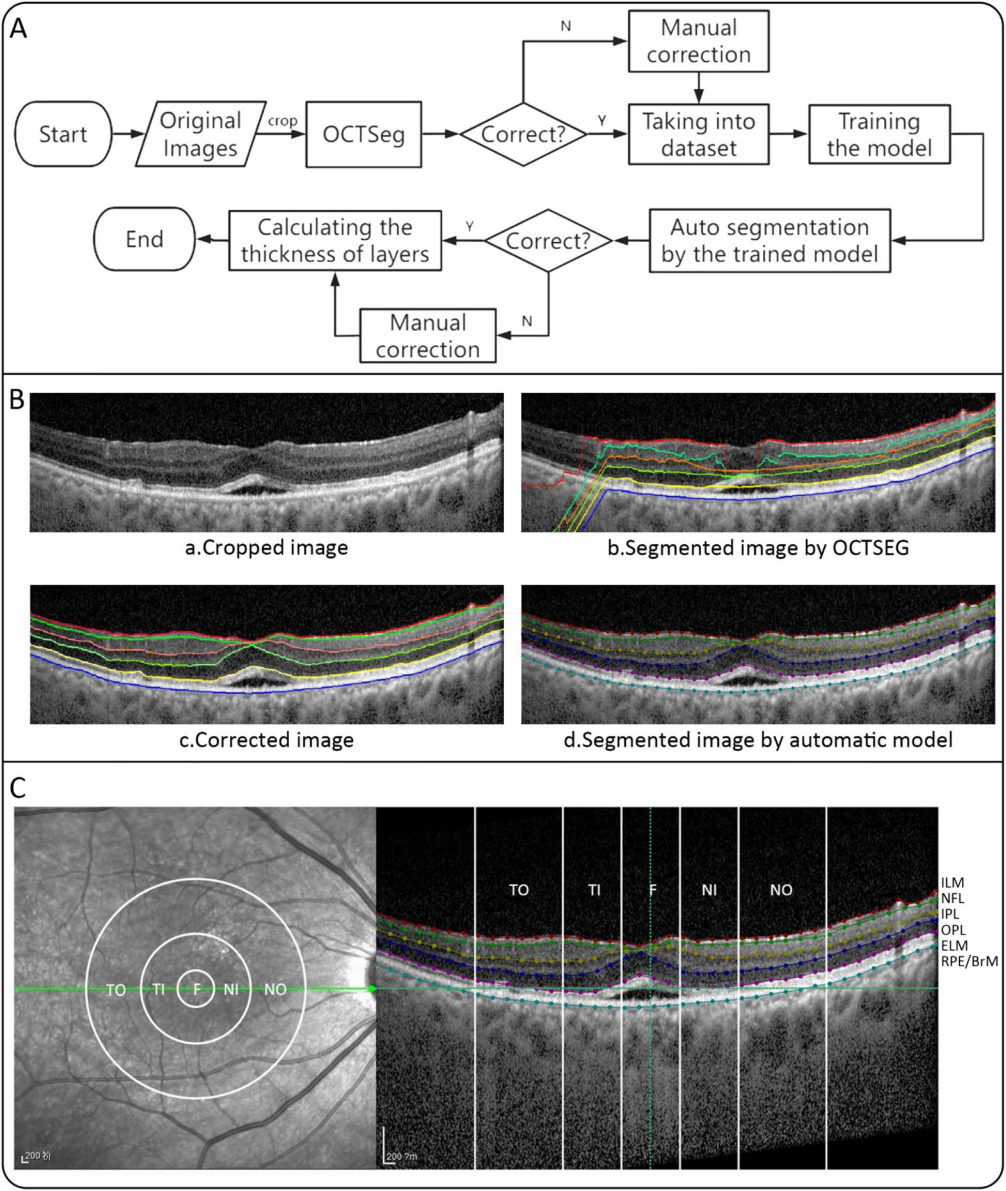
**A** Retinal segmentation procedure; **B** Example segmentation images of the OCT images; **C** Retinal segmentation and subregional instances

Regarding image processing, as shown in Fig. 1A, B, the OCT B-scans were initially cropped from original pairs of infrared reflectance (IR) and B-scan, with the size of 768 × 495. The initial segmentation of retinal layers was performed by the OCTSEG software (version 0.35) on 1670 OCT images, labeling the inner limiting membrane (ILM), NFL, inner plexiform layer (IPL), outer plexiform layer (OPL), external limiting membrane (ELM), and PRE/BrM [28]. The resulting images were divided into 5 distinct layers (Fig. 1C) defined as NFL (distance between the outer edge of ILM and the outer edge of NFL), GCL + IPL (distance between the outer edge of NFL and the outer edge of IPL), inner nuclear layer (INL) + OPL (distance between the outer edge of IPL and the outer edge of OPL), outer nuclear layer (ONL; distance between the outer edge of OPL and the outer edge of ELM), and ELM to RPE/BrM (distance between the outer edge of ELM and the outer edge of RPE or BrM). Then, due to the software limitations in the segmentation of OCT images with lesions, the above images were further manually corrected using the software Labelme (version 5.0.1). To reduce the workload of manual correction, the manually-corrected images were input into train a deep-learning-based OCT layering model [29], and the trained model predicted the layers of the remaining 6780 OCT images. We checked the predictions and manually corrected some wrongly-predicted images to obtain the final OCT layering labels. The thickness of each layer was obtained by calculating the distance between layers.

As we know that retinal thickness in different regions may change differently during the disease and may be affected by treatment. Besides, the layer thickness we calculated represents the average distance between layers, which may result in thickness differences that tend to converge toward the mean. To overcome this limitation, we divided the image into regions to enable a comparison of treatment effects across different regions by reference to the Treatment Diabetic Retinopathy Study (ETDRS) grid. As shown in Fig. 1C, we calculated the formula for converting microns to pixels based on the scale of the OCT image, and 5 regions 1,3 and 6 mm away from the center of the image were divided, namely nasal outer (NO), nasal inner (NI), fovea (F), temporal inner (TI), and temporal outer (TO), and then the thickness of each region before and after treatment was calculated.

### Statistical Analysis

For statistical description, mean ± standard deviation was used for all continuous variables and counts with percentages for categorical variables. The Shapiro–Wilk test was used to evaluate the normal distribution. Paired samples t-test was employed to compare the change of BCVA, CST, and individual retinal layers' thickness before and after treatment, and the LSD test was used for multiple comparisons. Pearson correlation coefficient (r) was used to evaluate the association between 2 variables. Statistical analyses were performed by using the Statistical Package for the Social Sciences software (IBM Corp. Released 2019. IBM SPSS Statistics for Windows, Version 26.0. Armonk, NY: IBM Corp). Probability values of P < 0.05 were considered indicative of statistical significance.

## Results

The study initially enrolled a total of 544 eyes from 496 patients, comprising 324 eyes with AMD and 220 eyes with PCV. The demographic and clinical characteristics of the study sample before treatment are shown in Table 1. In the AMD group, the mean age was 66.6 ± 10.9 years with 157 males and 167 females, and the mean VA and CST were 0.90 ± 0.64 LogMAR and 410.43 ± 191.60 μm respectively at baseline. The PCV group had a mean age of 64.4 ± 9.7 years with 120 males and 100 females, and mean VA and CST before treatment were 0.76 ± 0.61 LogMAR and 388.37 ± 188.51 μm respectively.

**Table 1:** Demographic characteristics of participants

Characteristic	AMD	PCV
Eyes	324	220
Age, y	66.6 ± 10.9	64.4 ± 9.7
Male/Female	157/167	120/100
Eyeside: L/R	174/150	118/102
BCVA(LogMAR)	0.90 ± 0.64	0.76 ± 0.61
CST, μm	410.43 ± 191.60	388.37 ± 188.51

*AMD* Age-related macular degeneration, *PCV* Polypoid choroidal angiopathy, *BCVA* Best corrected visual acuity, *logMAR* Logarithm of the minimum angle of resolution, *CST* Central subretinal thickness. Values are shown in mean ± SD, unless otherwise specified

## Retinal segmentation assessment

The relevant variables between pre-treatment and post-treatment of AMD and PCV patients were listed in Table 2. For the AMD group, BCVA, CST, and all retinal layers (NFL, GCL + IPL, INL + OPL, ONL, ELM to RPE/BrM) thickness of post-treatment showed a significant decrease compared to that of pre-treatment (*P* < 0.05). Meanwhile, in the PCV group, BCVA, CST, and the retinal layers of NFL, GCL + IPL, INL + OPL, and ELM to RPE/BrM were significantly decreased compared to the variates before treatment (*P* < 0.05). It is worth mentioning that, in both groups, the ELM to RPE/BrM layer was thicker than other layers before treatment (*P* < 0.05). But the decrease in ELM to RPE/BrM thickness after treatment was significantly greater than that of other layers (*P* < 0.001).

**Table 2:** Pre- and post-treatment in BCVA, CST and individual retinal layers of participants

Characteristic	AMD			PCV		
	**Pre**	**Post**	***P***	**Pre**	**Post**	***P***
BCVA(LogMAR)	0.90 ± 0.64	0.84 ± 0.64	**0.004**	0.76 ± 0.61	0.70 ± 0.59	**0.016**
CST, μm	410.43 ± 191.60	331.33 ± 152.72	** < 0.001**	388.37 ± 188.51	311.56 ± 142.38	** < 0.001**
NFL, μm	46.13 ± 7.92	45.21 ± 7.82	**0.006**	46.13 ± 8.92	45.08 ± 8.21	**0.007**
GCL + IPL, μm	64.60 ± 7.63	62.73 ± 8.00	** < 0.001**	63.98 ± 7.11	62.97 ± 7.08	** < 0.001**
INL + OPL, μm	57.97 ± 7.09	55.45 ± 7.46	** < 0.001**	57.50 ± 6.96	55.06 ± 6.08	** < 0.001**
ONL, μm	66.89 ± 17.83	62.49 ± 11.54	** < 0.001**	63.83 ± 14.92	62.79 ± 14.08	0.127
subELM, μm	125.42 ± 67.88	101.88 ± 41.02	** < 0.001**	127.20 ± 71.97	94.39 ± 33.70	** < 0.001**

*AMD* Age-related macular degeneration, *PCV* Polypoid choroidal angiopathy, *BCVA* Best corrected visual acuity, *logMAR* Logarithm of the minimum angle of resolution, *CST* Central subretinal thickness, *NFL* Nerve fiber layer, *GCL* Ganglion cell layer, *IPL* Inner plexiform layer, *INL* Inner nuclear layer, *OPL* Outer plexiform layer, *ONL* Outer nuclear layer, *ELM* External limiting membrane, *SubELM* ELM to RPE/BrM. Values are shown in mean ± SD, Values with statistical significance are in boldface

### Subregional assessment

When sorting out the data, we found that the lesions in most patients were not distributed in all regions of OCT images (Additional file 1), thus we conducted a subregional assessment to find out if there are any differences between individual layers (subregions as shown in Fig. 1(C)). The detailed results were presented in Additional file 2. For the AMD group, the thickness of ELM to RPE/BrM post-treatment showed a significant decrease compared to that of pre-treatment in each region, and the thickness of GCL + IPL, INL + OPL, and ONL in the NO, fovea, and TI region was significantly reduced. Similar findings were observed in the PCV group, that is, the ELM to RPE/BrM layer was significantly reduced in each region after treatment, and the thickness of most layers in the fovea region was significantly reduced, while no special rules were found in the changes of other layers in different regions. Multiple comparisons of the thickness changes within different regions obtained the same results in two groups, that is, the thickness reduction of ELM to RPE/BrM layers was significantly greater than that of other layers in each region (*P* < 0.001).

**Fig. 2:**
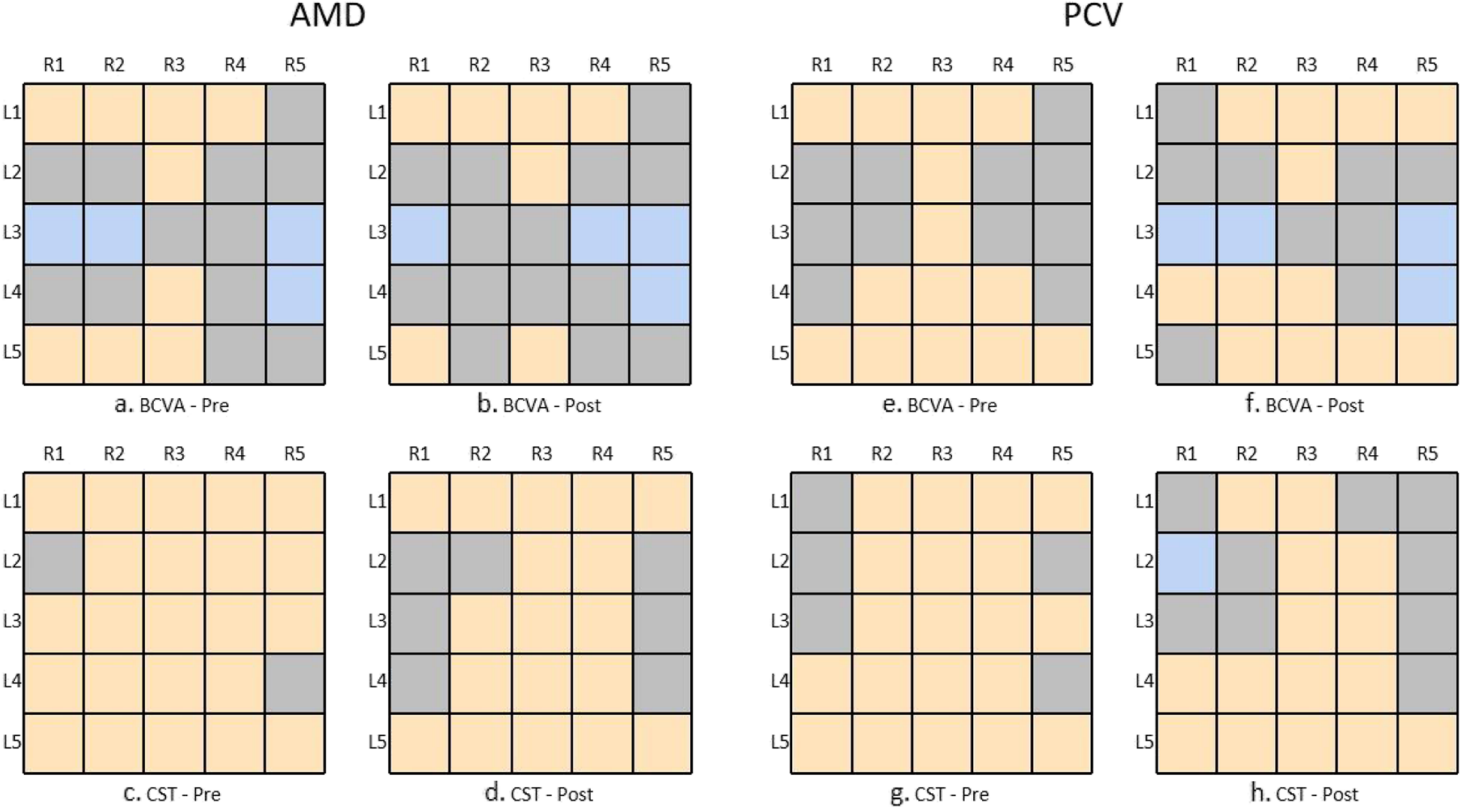
Correlation between BCVA/CST and thickness of individual regions before and after treatment. Yellow indicates a significant positive correlation, blue indicates a significant negative correlation, and gray indicates that there is no linear correlation. L1 = NFL, L2 = GCL + IPL, L3 = INL + OPL, L4 = ONL, L5 = ELM to RPE/BrM; R1 = Nasal Outer, R2 = Nasal Inner, R3 = Fovea, R4 = Temporal Inner, R5 = Temporal Outer

In addition, we used the Spearman correlation coefficient (R) to evaluate the association between regional thickness and BCVA/CST before and after treatment, as shown in Fig. 2. For the AMD group, at baseline, BCVA was positively correlated with 9 regions and negatively correlated with 4 regions. The region with the highest correlation was the fovea region in the NFL layer with R = 0.252, *p* < 0.001. The results after treatment were similar to those before treatment, and the region with the highest correlation remained the fovea region in NFL with R = 0.307, *p* < 0.001. CST was positively related to most regions before and after treatment. The region with the highest correlation before treatment was the fovea region in ELM to RPE/BrM with R = 0.727, *p* < 0.001. The region with the highest correlation after treatment remained the fovea region in ELM to RPE/BrM, with R = 0.702, *p* < 0.001. The results of the PCV group were similar to those of the AMD group. For BCVA before and after treatment, the fovea region in NFL was the most correlated, while for CST before and after treatment, the fovea region in ELM to RPE/BrM was the most correlated. The correlation analysis results were shown in Additional files 3 and 4.

## Discussion

The present study demonstrated a statistically significant reduction in CST and individual retinal layers in patients with AMD or PCV following treatment with anti-VEGF agents, along with a significant improvement in visual acuity compared to baseline. Notably, ELM to RPE/BrM was often thicker than other layers before treatment, which may be attributed to the fact that lesions in AMD or PCV tend to occur in the RPE layer, while the reduction of the RPE layer was significantly greater than that in other layers after treatment. These results may indicate that anti-VEGF treatment had a better therapeutic effect on edema sites.

Our findings about the layer changes were in accordance with previous studies, elaborating improved visual acuity and reduced retinal thickness in whole or individual layers following treatment with anti-VEGF agents [12, 22, 30]. It is worth mentioning that previous clinical studies on retinal alterations after intravitreal injection of anti-VEGF mainly reported changes in NFL or GCL-IPL evaluated by OCT [23, 24, 31], just with a relatively small sample size as well [32]. However, our study analyzed 545 eyes of 497 patients, meanwhile, and examined individual inner and outer retinal layers, with a comparative assessment of therapeutic effects.

Moreover, to eliminate the fact that the average thickness calculated may lead to differences tending to converge towards the mean, we divided OCT images into regions centered on the macula and calculated the thickness of each retina layer in different regions. To our knowledge, this is the first study to investigate the change in the different regions after anti-VEGF treatment using OCT images. We observed a reduction in the thickness of ELM to RPE/BrM in each region following treatment, while the thickness of other layers did not decrease significantly in every region. The decrease in ELM-RPE/BrM was significantly greater than that of other layers in every region, suggesting that anti-VEGF agents are indeed more effective on the ELM to RPE/BrM.

We also conducted a correlation analysis between BCVA and the thickness of individual regions and obtained similar results before and after treatment, where BCVA was positively correlated with most foveal regions, and NFL had the highest correlation. Although the correlation coefficient between thickness and BCVA is not large enough, it can be explained that the change in fovea thickness has a certain impact on visual acuity to some extent. The thickness of most regions is positively correlated with CST, and the correlation between ELM to RPE/BrM and CST is the largest, which verifies our previous view that the lesions mostly occur in ELM to RPE/BrM. The correlation between BCVA and the thickness of regions after treatment suggests that the fovea region thickness may affect more on visual outcome. The therapeutic effect of anti-VEGF agents in ELM to RPE/BrM and foveal region may suggest that precise regional injection may be more effective in restoring in the future. For example, if anti-VEGF treatments inject into edema sites or near the macular fovea, they may be more effective in restoring vision.

There are some limitations to this paper, the software we used divided the retina into 6 layers, which may underestimate the impact of individual retinal layer thickness changes on diseases or treatments. Increasing the number of layers could provide a more detailed analysis of different layer thicknesses to generate more specific results. Deep learning algorithms applied in the automatic segmentation of patients’ OCT images provide the feasibility of further analysis. However, the data amount can affect the segmentation results, we need more publicly available labeled patients’ datasets for more analytical research, which may be helpful for future precise treatment. Additionally, the foveal region depicted the most significant decrease in ELM to RPE/BrM layer, while other layers had the largest decline with a P-value greater than 0.05, which may not be statistically significant due to insufficient sample size. Moreover, the study followed up for only 6 months, with limited follow-up numbers. Extending the outcome time could furnish more realistic results on alterations in visual acuity and OCT. Lastly, further stratified and subregional analysis can be carried out on other diseases requiring anti-VEGF treatment in the dataset, which may obtain similar results to prove the effect of anti-VEGF treatment. This could be a start of a precision layer or region injection for retina-related diseases.

## Conclusion

In conclusion, we labeled a large number of retinal layers of OCT images with AMD and PCV. We found a significant reduction in retinal layer thickness based on the dataset. We also proposed a subregional assessment of changes after treatments and find different associations between subregional layers and BCVA/CST. The thickness of ELM to RPE/BrM decreased the greatly in every region, suggesting that anti-VEGF treatment targeted edema sites better.

### Supplementary Information

**Additional file 1:** ** Supplementary** **Figure 1.** lesions distributed regions in patients with AMD and PCV: (A) AMD patient with lesions concentrated in the fovea region; (B) AMD patient with lesions concentrated outside the fovea; (C) PCV patient with lesions concentrated in the fovea region; (D) PCV patient with lesions concentrated outside the fovea.

**Additional file 2:** **Supplementary Table 1.** Pre- and post-treatment in different regions of individual retinal layers of participants.

**Additional file 3:** **Supplementary Table 2.** Correlation coefficient (R) between BCVA and thickness of individual regions before and after treatment.

**Additional file 4:** **Supplementary Table 3.** Correlation coefficient (R) between CST and thickness of individual regions before and after treatment.

## Data Availability

The datasets during and analyzed in the current study are available from the corresponding author upon reasonable request.

## Abbreviations

AMD: Age-related macular degeneration
BCVA: Best corrected visual acuity
BrM: Bruch membrane
CME: Cystoid macular edema
CNV: Choroidal neovascularization
CST: Central subretinal thickness
DME: Diabetic macular edema
DR: Diabetic retinopathy
ELM: External limiting membrane
ETDRS: Treatment Diabetic Retinopathy Study
F: Fovea
GCL: Ganglion cell layer
ILM: Inner limiting membrane
INL: Inner nuclear layer
IPL: Inner plexiform layer
IR: Infrared reflectance
logMAR: Logarithm of the minimum angle of resolution
NFL: Nerve fiber layer
NI: Nasal inner
NO: Nasal outer
OCT: Optical coherence tomography
ONL: Outer nuclear layer
OPL: Outer plexiform layer
PCV: Polypoid choroidal angiopathy
PRE: Retinal pigment epithelium
ROP: Retinopathy of prematurity
RVO: Retinal vein occlusion
TI: Temporal inner
TO: Temporal outer
VEGF: Vascular endothelial growth factor

## Footnotes

1 https://tianchi.aliyun.com/specials/promotion/APTOS?spm=a2c22.12281978.0.0

## References

[1] Heier JS, Brown DM, Chong V (2012). Intravitreal aflibercept (VEGF trap-eye) in wet age-related macular degeneration. Ophthalmology.

[2] Penn JS, Madan A, Caldwell RB (2008). Vascular endothelial growth factor in eye disease. Prog Retin Eye Res.

[3] Campochiaro PA, Akhlaq A (2021). Sustained suppression of VEGF for treatment of retinal/choroidal vascular diseases. Prog Retin Eye Res.

[4] Das A, McGuire PG (2003). Retinal and choroidal angiogenesis: pathophysiology and strategies for inhibition. Prog Retin Eye Res.

[5] Falavarjani KG, Nguyen QD (2013). Adverse events and complications associated with intravitreal injection of anti-VEGF agents: a review of literature. Eye (Lond).

[6] Wallsh JO, Gallemore RP (2021). Anti-VEGF-resistant retinal diseases: a review of the latest treatment options. Cells.

[7] Mettu PS, Allingham MJ, Cousins SW (2021). Incomplete response to Anti-VEGF therapy in neovascular AMD: Exploring disease mechanisms and therapeutic opportunities. Prog Retin Eye Res.

[8] Chakravarthy U, Harding SP, Rogers CA (2013). Alternative treatments to inhibit VEGF in age-related choroidal neovascularisation: 2-year findings of the IVAN randomised controlled trial. Lancet.

[9] Wong WL, Su X, Li X (2014). Global prevalence of age-related macular degeneration and disease burden projection for 2020 and 2040: a systematic review and meta-analysis. Lancet Glob Health.

[10] Mitchell P, Liew G, Gopinath B (2018). Age-related macular degeneration. Lancet.

[11] Bo Q, Zhang M, Chen J (2023). Progression of polypoidal lesions associated with exudative recurrence in polypoidal choroidal vasculopathy. Ophthalmology.

[12] Fenner BJ, Ting DSW, Tan ACS (2020). Real-world treatment outcomes of age-related macular degeneration and polypoidal choroidal vasculopathy in Asians. Ophthalmol Retina.

[13] Cheung CMG, Lai TYY, Ruamviboonsuk P (2018). Polypoidal choroidal vasculopathy: definition, pathogenesis, diagnosis, and management. Ophthalmology.

[14] Laíns I, Wang JC, Cui Y (2021). Retinal applications of swept source optical coherence tomography (OCT) and optical coherence tomography angiography (OCTA). Prog Retin Eye Res.

[15] Kanagasingam Y, Bhuiyan A, Abràmoff MD (2014). Progress on retinal image analysis for age-related macular degeneration. Prog Retin Eye Res.

[16] Cheung CMG, Lai TYY, Teo K (2021). Polypoidal choroidal vasculopathy: consensus nomenclature and non-indocyanine green angiograph diagnostic criteria from the Asia-Pacific ocular imaging society PCV workgroup. Ophthalmology.

[17] Sacconi R, Fragiotta S, Sarraf D (2023). Towards a better understanding of non-exudative choroidal and macular neovascularization. Prog Retin Eye Res.

[18] Trinh M, Kalloniatis M, Alonso-Caneiro D (2022). High-density optical coherence tomography analysis provides insights into early/intermediate age-related macular degeneration retinal layer changes. Invest Ophthalmol Vis Sci.

[19] Zucchiatti I, Parodi MB, Pierro L (2015). Macular ganglion cell complex and retinal nerve fiber layer comparison in different stages of age-related macular degeneration. Am J Ophthalmol.

[20] Muftuoglu IK, Ramkumar HL, Bartsch DU (2018). Quantitative analysis of the inner retinal layer thicknesses in age-related macular degeneration using corrected optical coherence tomography segmentation. Retina.

[21] Borrelli E, Barresi C, Lari G, Berni A, Battista M, Reibaldi M, Cascavilla ML, Bandello F (2023). Capturing the transition from intermediate to neovascular AMD: longitudinal inner retinal thinning and factors associated with neuronal loss. Invest Ophthalmol Vis Sci.

[22] Moon DRC, Lee DK, Kim SH (2015). Aflibercept treatment for neovascular age-related macular degeneration and polypoidal choroidal vasculopathy refractory to anti-vascular endothelial growth factor. Korean J Ophthalmol.

[23] Zivkovic M, Radosavljevic A, Zlatanovic M (2023). Influence of multiple anti-VEGF injections on retinal nerve fiber layer and ganglion cell-inner plexiform layer thickness in patients with exudative age-related macular degeneration. Medicina (Kaunas).

[24] Kim SY, Yoon MH, Chin HS (2020). Changes in the ganglion cell-inner plexiform layer after consecutive intravitreal injections of anti-vascular endothelial growth factor in age-related macular degeneration patients. Korean J Ophthalmol.

[25] Wood A, Binns A, Margrain T (2011). Retinal and choroidal thickness in early age-related macular degeneration. Am J Ophthalmol.

[26] Resch MD, Balogh A, Kurth T (2022). Atrophy of retinal vessels in neovascular age-related macular degeneration following long-term treatment with 20 intravitreal anti-VEGF injections. BMC Ophthalmol.

[27] von der Emde L, Vaisband M, Hasenauer J (2022). Histologic cell shape descriptors for the retinal pigment epithelium in age-related macular degeneration: a comparison to unaffected eyes. Transl Vis Sci Technol.

[28] Mayer MA, Hornegger J, Mardin CY (2010). Retinal nerve fiber layer segmentation on fd-oct scans of normal subjects and glaucoma patients. Biomed Opt Express.

[29] Qiu Z, Hu Y, Zhang J (2022). FGAM: a pluggable light-weight attention module for medical image segmentation. Comput Biol Med.

[30] Lu Y, Huang W, Zhang Y (2021). Factors for visual acuity improvement after Anti-VEGF treatment of wet age-related macular degeneration in China: 12 months follow up. Front Med (Lausanne).

[31] Valverde-Megías A, Ruiz-Calvo A, Murciano-Cespedosa A (2019). Long-term effect of intravitreal ranibizumab therapy on retinal nerve fiber layer in eyes with exudative age-related macular degeneration. Graefes Arch Clin Exp Ophthalmol.

[32] Wichrowska M, Liberski S, Rzeszotarska A (2022). Examination of inner retinal layers in unilateral wet age-related macular degeneration treated with Anti-VEGF, compared to fellow untreated eyes. Int J Mol Sci.

