# Supplementary material for: Retinal layers changes in patients with age-related macular degeneration treated with intravitreal anti-VEGF agents
Source: BMC Ophthalmol. 2023 Nov 13;23:451. doi: 10.1186/s12886-023-03203-w (PMC10642061; doi:10.1186/s12886-023-03203-w)
Supplement: Supplementary file 1 — Additional file 1: Supplementary Figure 1. lesions distributed regions in patients with AMD and PCV: (A) AMD patient with lesions concentrated in the fovea region; (B) AMD patient with lesions concentrated outside the fovea; (C) PCV patient with lesions concentrated in the fovea region; (D) PCV patient with lesions concentrated outside the fovea. [file 12886_2023_3203_MOESM1_ESM.pdf]

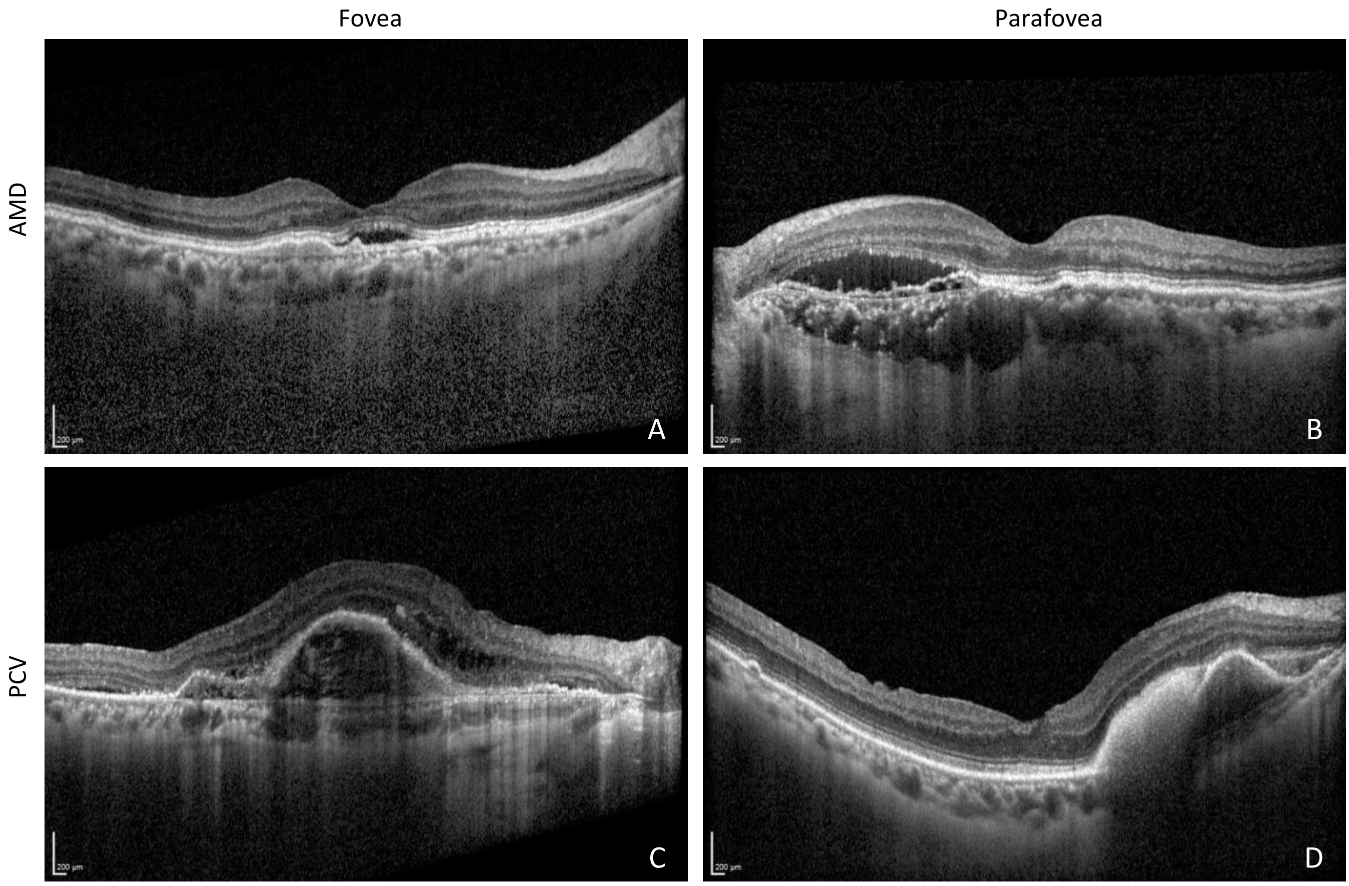

Supplementary Figure 1. lesions distributed regions in patients with AMD and PCV: (A) AMD patient with lesions concentrated in the fovea region; (B) AMD patient with lesions concentrated outside the fovea; (C) PCV patient with lesions concentrated in the fovea region; (D) PCV patient with lesions concentrated outside the fovea.
