# Supplementary material for: Retinal layers changes in patients with age-related macular degeneration treated with intravitreal anti-VEGF agents
Source: BMC Ophthalmol. 2023 Nov 13;23:451. doi: 10.1186/s12886-023-03203-w (PMC10642061; doi:10.1186/s12886-023-03203-w)
Supplement: Supplementary file 2 — Additional file 2: Supplementary Table 1. Pre- and post-treatment in different regions of individual retinal layers of participants. [file 12886_2023_3203_MOESM2_ESM.pdf]

Supplementary Table 1. Pre- and post-treatment in different regions of individual retinal layers of participants

Characteristic		AMD			PCV		
		**Pre, μm**	**Post, μm**	**P**	**Pre, μm**	**Post, μm**	**P**
NO	NFL	49.74±16.30	48.86±15.78	0.32	47.89±14.10	46.36±13.00	**0.07**
	GCL+IPL	80.78±15.01	79.05±14.42	**0.031**	82.18±12.44	82.37±12.49	0.812
	INL+OPL	68.70±14.33	65.90±13.76	**<0.001**	69.89±12.56	67.00±11.3	**<0.001**
	ONL	64.60±23.97	61.25±17.82	**0.016**	63.81±18.49	65.58±46.17	0.507
	subELM	114.95±84.75	94.59±53.37	**<0.001**	119.71±106.51	92.07±54.24	**<0.001**
NI	NFL	32.75±11.54	32.49±11.48	0.708	31.63±9.84	30.36±7.32	0.082
	GCL+IPL	85.70±20.26	83.58±19.30	0.066	86.97±20.79	84.47±20.71	**0.047**
	INL+OPL	75.79±21.07	73.52±21.27	0.093	76.93±18.21	72.06±17.05	**<0.001**
	ONL	79.16±52.16	69.32±28.27	**0.001**	73.77±45.93	75.36±61.08	0.61
	subELM	180.47±141.38	140.32±106.43	**<0.001**	173.96±134.15	123.69±86.80	**<0.001**
F	NFL	27.23±10.65	26.33±9.69	0.122	26.45±8.27	23.77±6.90	**<0.001**
	GCL+IPL	53.52±29.22	48.65±27.45	**0.005**	50.83±29.94	44.76±24.20	**0.003**
	INL+OPL	56.68±25.26	52.43±26.34	**0.014**	55.25±32.74	50.09±24.12	**0.03**
	ONL	87.42±74.21	72.42±39.85	**<0.001**	74.50±53.01	76.82±57.45	0.417
	subELM	226.70±160.47	168.24±122.57	**<0.001**	224.45±167.58	150.59±98.32	**<0.001**
TI	NFL	29.38±14.08	29.53±9.15	0.851	29.33±7.96	28.21±5.83	0.071
	GCL+IPL	78.76±21.42	76.31±20.39	**0.032**	79.90±19.46	75.62±19.31	**0.001**
	INL+OPL	70.41±20.00	64.39±17.86	**<0.001**	69.38±18.60	63.86±16.29	**<0.001**
	ONL	74.14±41.94	69.61±32.86	**0.044**	71.37±41.02	69.59±28.46	0.48
	subELM	182.74±138.44	134.21±93.1	**<0.001**	179.2±143.17	125.08±91.79	**<0.001**
TO	NFL	28.99±6.58	28.46±6.26	0.181	28.92±6.11	28.18±6.11	0.154
	GCL+IPL	71.70±14.77	69.39±14.82	**0.003**	71.53±12.87	70.31±13.04	0.169
	INL+OPL	62.16±12.81	58.30±11.33	**<0.001**	61.33±16.03	58.73±11.13	**0.022**
	ONL	64.67±19.45	63.71±17.45	0.388	64.85±24.86	62.15±15.65	0.095
	subELM	122.27±100.73	94.71±55.41	**<0.001**	126.68±104.10	99.60±60.82	**<0.001**

AMD = Age-related macular degeneration; PCV = Polypoid choroidal angiopathy; BCVA = Best corrected visual acuity; logMAR = Logarithm of the minimum angle of resolution; CST = Central subretinal thickness; NFL = Nerve fiber layer; GCL = Ganglion cell layer; IPL = Inner plexiform layer; INL = Inner nuclear layer; OPL = Outer plexiform layer; ONL = Outer nuclear layer; ELM = External limiting membrane; SubELM = ELM to RPE/BrM; NO = nasal outer; NI = nasal inner; F = fovea; TI = temporal inner; TO = temporal outer. Values are shown in mean ± SD. Values with statistical significance are in boldface.
