# Supplementary material for: Retinal layers changes in patients with age-related macular degeneration treated with intravitreal anti-VEGF agents
Source: BMC Ophthalmol. 2023 Nov 13;23:451. doi: 10.1186/s12886-023-03203-w (PMC10642061; doi:10.1186/s12886-023-03203-w)
Supplement: Supplementary file 3 — Additional file 3: Supplementary Table 2. Correlation coefficient (R) between BCVA and thickness of individual regions before and after treatment. [file 12886_2023_3203_MOESM3_ESM.pdf]

Supplementary Table 2. Correlation coefficient (R) between BCVA and thickness of individual regions before and after treatment

	**AMD**					**PCV**				
	**NO**	**NI**	**F**	**TI**	**TO**	**NO**	**NI**	**F**	**TI**	**TO**
	**Pre-treatment**									
NFL	0.196**	0.281**	0.252**	0.122*	0.066	0.148*	0.269**	0.375**	0.224**	0.104
GCL+IPL	-0.051	-0.076	0.182**	0.043	-0.049	0.000	0.013	0.267**	0.090	-0.009
INL+OPL	-0.115*	-0.154**	0.080	-0.021	-0.198**	-0.002	-0.022	0.246**	0.053	0.006
ONL	-0.101	0.082	0.132*	0.022	-0.185**	0.098	0.221**	0.193**	0.176**	-0.030
subELM	0.205**	0.230**	0.140*	0.078	0.089	0.187**	0.301**	0.352**	0.286**	0.200**
	**Post-treatment**									
NFL	0.152**	0.200**	0.307**	0.235**	0.099	0.108	0.244**	0.300**	0.199**	0.203**
GCL+IPL	-0.102	-0.013	0.259**	0.042	-0.042	-0.069	-0.079	0.233**	0.001**	-0.041
INL+OPL	-0.216**	-0.074	0.017	-0.131*	-0.206**	-0.236**	-0.193**	0.089	-0.101	-0.143*
ONL	-0.105	0.012	-0.014	-0.058	-0.182**	0.150*	0.254**	0.191**	0.104	-0.188**
subELM	0.117*	0.106	0.109*	0.052	-0.044	0.108	0.214**	0.343**	0.254**	0.148*

AMD = Age-related macular degeneration; PCV = Polypoid choroidal angiopathy; BCVA = Best corrected visual acuity; logMAR = Logarithm of the minimum angle of resolution; CST = Central subretinal thickness; NFL = Nerve fiber layer; GCL = Ganglion cell layer; IPL = Inner plexiform layer; INL = Inner nuclear layer; OPL = Outer plexiform layer; ONL = Outer nuclear layer; ELM = External limiting membrane; SubELM = ELM to RPE/BrM; NO = nasal outer; NI = nasal inner; F = fovea; TI = temporal inner; TO = temporal outer. Values are shown in mean ± SD. *=statistically significant at $p<0.05$ level. **=statistically significant at $p<0.001$ level.
