# Supplementary material for: Retinal layers changes in patients with age-related macular degeneration treated with intravitreal anti-VEGF agents
Source: BMC Ophthalmol. 2023 Nov 13;23:451. doi: 10.1186/s12886-023-03203-w (PMC10642061; doi:10.1186/s12886-023-03203-w)
Supplement: Supplementary file 4 — Additional file 4: Supplementary Table 3. Correlation coefficient (R) between CST and thickness of individual regions before and after treatment. [file 12886_2023_3203_MOESM4_ESM.pdf]

Supplementary Table 3. Correlation coefficient (R) between CST and thickness of individual regions before and after treatment

	AMD					PCV				
	**NO**	**NI**	**F**	**TI**	**TO**	**NO**	**NI**	**F**	**TI**	**TO**
	**Pre-treatment**									
NFL	0.210**	0.282**	0.308**	0.134*	0.173**	0.130	0.290**	0.527**	0.263**	0.269**
GCL+IPL	0.061	0.127*	0.366**	0.278**	0.120*	-0.034	0.183**	0.394**	0.336**	0.120
INL+OPL	0.125*	0.192**	0.370**	0.358**	0.150**	0.132	0.179**	0.227**	0.420**	0.241**
ONL	0.125*	0.311**	0.344**	0.263**	-0.006	0.168*	0.354**	0.331**	0.245**	0.006
subELM	0.288**	0.604**	0.727**	0.635**	0.415**	0.179**	0.532**	0.718**	0.509**	0.259**
	**Post-treatment**									
NFL	0.191**	0.275**	0.426**	0.202**	0.137*	0.055	0.177**	0.484**	0.076	0.030
GCL+IPL	-0.075	0.091	0.509**	0.331**	0.011	-0.133*	0.014	0.531**	0.178**	-0.038
INL+OPL	0.012	0.255**	0.461**	0.321**	0.035	-0.061	0.114	0.362**	0.137*	-0.063
ONL	-0.049	0.211**	0.253**	0.239**	0.057	0.157*	0.388**	0.414**	0.228**	-0.099
subELM	0.239**	0.548**	0.702**	0.479**	0.203**	0.217**	0.495**	0.683**	0.385**	0.151*

AMD = Age-related macular degeneration; PCV = Polypoid choroidal angiopathy; BCVA = Best corrected visual acuity; logMAR = Logarithm of the minimum angle of resolution; CST = Central subretinal thickness; NFL = Nerve fiber layer; GCL = Ganglion cell layer; IPL = Inner plexiform layer; INL = Inner nuclear layer; OPL = Outer plexiform layer; ONL = Outer nuclear layer; ELM = External limiting membrane; SubELM = ELM to RPE/BrM; NO = nasal outer; NI = nasal inner; F = fovea; TI = temporal inner; TO = temporal outer. Values are shown in mean ± SD. *=statistically significant at p<0.05 level. **=statistically significant at p<0.001 level.
